# Representing Geospatial Environment Observation Capability Information: A Case Study of Managing Flood Monitoring Sensors in the Jinsha River Basin

**DOI:** 10.3390/s16122144

**Published:** 2016-12-16

**Authors:** Chuli Hu, Qingfeng Guan, Jie Li, Ke Wang, Nengcheng Chen

**Affiliations:** 1 Faculty of Information Engineering, China University of Geosciences (Wuhan), Wuhan 430074, China; guanqf@cug.edu.cn (Q.G.); harrypeas@me.com (J.L.); wmiller1978@whu.edu.cn (K.W.); 2 National Engineering Research Center for Geographic Information System, China University of Geosciences (Wuhan), Wuhan 430074, China; 3 State Key Lab for Information Engineering in Surveying, Mapping and Remote Sensing, Wuhan University, Wuhan 430079, China; cnc@whu.edu.cn; 4 Collaborative Innovation Center of Geospatial Technology, Wuhan 430079, China

**Keywords:** environment monitoring, Sensor Web, observation capability representation, sensor discovery and collaboration, flood

## Abstract

Sensor inquirers cannot understand comprehensive or accurate observation capability information because current observation capability modeling does not consider the union of multiple sensors nor the effect of geospatial environmental features on the observation capability of sensors. These limitations result in a failure to discover credible sensors or plan for their collaboration for environmental monitoring. The Geospatial Environmental Observation Capability (GEOC) is proposed in this study and can be used as an information basis for the reliable discovery and collaborative planning of multiple environmental sensors. A field-based GEOC (GEOCF) information representation model is built. Quintuple GEOCF feature components and two GEOCF operations are formulated based on the geospatial field conceptual framework. The proposed GEOCF markup language is used to formalize the proposed GEOCF. A prototype system called GEOCapabilityManager is developed, and a case study is conducted for flood observation in the lower reaches of the Jinsha River Basin. The applicability of the GEOCF is verified through the reliable discovery of flood monitoring sensors and planning for the collaboration of these sensors.

## 1. Introduction

### 1.1. Discovery of Earth Environmental Sensors under the Sensor Web Environment

The Sensor Web is an emerging paradigm for integrating multiple satellites, in situ sensors, and data systems into a common infrastructure that enables the interoperable usage of sensor metadata, data, and data products over the internet [1,2]. The functionality required from such infrastructure includes sensor discovery, access, tasking, eventing, and alerting [3]. The Sensor Web Enablement (SWE), developed by the Open Geospatial Consortium (OGC), defines a standard framework to realize the Sensor Web notion [4]. The Global Earth Observation System of Systems (GEOSS) [5], which combines the advantages of Sensor Web technology, aims to integrate heterogeneous sensors and data systems across institutional and political boundaries to continuously monitor the state of the Earth. Along with the development of geospatial web services [6], distributed geospatial environmental sensors and their observations have been registered and stored in a geospatial sensor clearinghouse using standards-based web service interfaces. The interfaces include sensor observation services and the Catalog Service for the Web (CSW), which enables the web-based discovery of environmental sensors [7]. Specifically, when an emergency observation is requested, inquirers can openly and standardly retrieve a list of environmental sensors through a web-based platform under the Sensor Web environment. 

The Sensor Web mechanism has been widely established across different fields, such as wildfire [8], atmosphere [9], soil moisture [10], and tsunami [11] studies. Recently, extensive efforts have been applied to the SWE and GEOSS platforms. For example, the European Sensors Anywhere (SANY) and Open architecture for Smart and Interoperable networks in Risk management based on In-situ Sensors (OSIRIS) projects [12,13] used the SWE standard service interface to manage air pollution monitoring by associating in situ sensors with sensor networks. The Namibia Sensor Web project [14,15] successfully integrated the National Aeronautics and Space Administration (NASA) Earth Observing-1 (EO-1) satellite data and other satellite data sets to establish a flood early warning system. The NASA Sensor Web 2.0 project [16] automatically combined NASA’s Terra, Aqua, and EO-1 satellites with an Unmanned Aerial System in a wildfire detection scenario. The GEOSS Architecture Implementation Pilot deployed the GEOSS Common Infrastructure provided core capabilities that enabled sensor web resources (sensor systems, data, and products) to be registered, discovered, understood, and accessed by users and decision-makers [17]. GEOSS enables the comprehensive, coordinated, and sustained observations of the Earth and includes nine societal benefit areas: Agriculture, Biodiversity, Climate, Disasters, Ecosystems, Energy, Health, Water, and Weather. The Sensor Web technology is a widely-accepted means for Earth environmental monitoring [3,18,19]. Sensor discovery is the core functionality of the Sensor Web [20], and the reliable and collaborative discovery of various environmental sensors is the premise to ensure the authenticity of sensor-observed data and the follow-up applications for environmental monitoring. However, most applications or projects have focused on sensor data access, data processing, or product production and delivery after the given sensors have been web-based tied together. The source of those sensors often did not undergo the discovery process from the sensor clearinghouse. In most cases, the mode to select those sensors was the “setting” mode provided by the project requirements, instead of the on-demand “discovery” mode matched from the sensor clearinghouse. Liang et al. [21] noted that the sensor discovery on the Sensor Web remains a challenge and stated that the full potential of the Sensor Web has not yet been realized. In this context, this work focuses on the reliable and integrated discovery of environmental sensors.

### 1.2. Representation of Observation Capability Information

Considerable attention has been directed toward representing the observation capabilities of environmental sensors. The quantitative representation of observation capability concentrates on the following aspects: spatial coverage [22], revisit time [23], and a specific satellite observation task [24]. However, studies on quantitative observation capability representation are typically attached to different observation missions. Namely, one numerical observation capability value for a distinct application usage cannot be transferred to other observation missions. In addition, a quantitative observation capability value does not contain the rich observation information that is required for decision-making when using a collaboration of multiple environmental sensors. Since 2007, the representation of the observation capability from the information modeling viewpoint has been regarded as the core research objective of the SWE [10,25,26,27]. The SWE defines a standard framework that aims to enable the interoperable use of sensors [3]. The Sensor Model Language (SensorML) is part of the SWE framework information model. Hu et al. [25] established a SensorML-based observation capability metadata model for Earth observation sensor discovery. Fan et al. [10] further proposed a sensor capability representation model for remote sensors that are used for soil moisture monitoring. The above studies proposed capability representation models that adopted SensorML as the description carrier and focused primarily on the static and inherent observation capability properties of a single sensor. Although the static and inherent observation capability properties of a single sensor have been determined [25], a representation model that considers the integration of multiple sensor observation capabilities is still lacking. This limitation has resulted in a failure to plan for the collaboration of these sensors for environmental monitoring.

### 1.3. Representation Requirements of Geospatial Environmental Observation Capability Information

In addition to supplementing new observation equipment, fully utilizing existing sensors is key to improving the observation of the global earth environment. Thus, a more integrated and systematic understanding of the capabilities of earth observation sensors is essential [17,28]. Owing to the diversity and complexity of environmental disasters, collaborative observations from multiple sensors for environmental monitoring is necessary [29]. The capability of integrated observations generated via the union of multiple sensors should be determined. After the union of two or more sensors, the new compounded capability of observations should explicitly present the association of the capabilities of the independent sensors.

Every sensor is deployed in a practical geospatial environment; thus, its observation efficiency is affected by geospatial environmental features (e.g., illumination, cloud coverage, and terrain). The current sensor observation information models do not consider the influence of geospatial environmental features [25]. In ideal weather conditions, the Moderate Resolution Imaging Spectroradiometer (MODIS) aboard the Terra satellite crosses the monitoring area at the specified time and can theoretically complete the task of observing the target phenomenon. However, the local environment can be covered by clouds when the satellite passes. In this case, the sensor (MODIS) is unreliable for the specified observation task. Geospatial environmental features are essential factors that can constrain the observation capability of satellite sensors.

In geographic information science, the geospatial environment is a large-scale space that may be represented by different data models from different perspectives [30]. Under the conceptual framework of a geospatial data model, identifiable objects, such as mountains and land parcels, can be represented using an object model, whereas continuous and amorphous phenomena can be demonstrated using a field model. Theoretically, a geospatial field can be viewed as a mapping between a locational reference frame and an attribute domain [31,32]. A formal field model has been expressed by Goodchild [33] as <*x*, *z*(*x*)>, where *x* is one point of a continuous location, and *z*(*x*) is the value of property *Z* at point *x*. Numerous geospatial entities (e.g., points, lines, polygons, and grid cells) have been represented in object-oriented models [34], but many geospatial environmental features, such as digital elevation models [35], temperature [36], and wind [37], have also been modeled from the field perspective. Compared with an object model, a field model is a more basic model representing geographical phenomena that facilitates the objective cognition of geographical phenomena [31]. 

In this study, we propose a Field-based Geospatial Environmental Observation Capability (GEOCF) information representation model that considers the observation capability information of the environmental sensors to be the source and the features (e.g., cloud and topography) of a geographical observation environment to be the constraints. The proposed GEOCF information representation model is applied to determine the accurate discovery and efficient planning of flood sensors in the Jinsha River Basin. The remainder of this paper is organized as follows: Section 2 elaborates on the GEOCF information representation model. Section 3 presents the application and results. Section 4 lists the characteristics of the GEOCF information representation model based on the discussion. Section 5 presents the conclusions and the directions for future work.

## 2. Representation Model of Geospatial Environmental Observation Capability Information

### 2.1. Space Abstraction in the Model

Observation capability information can be represented from two perspectives: a discrete sensor object and a continuous spatial field. The observation capability of a discrete sensor object is confined to a single sensor's static and inherent observation properties, whereas continuous spatial observation capability information provides the dynamic observation capability information from a combination of multiple sensors. 

Selecting the appropriate geospatial modeling perspective is important in understanding geographical phenomena [32]. The geospatial field modeling concept originates from classical physics and is used to represent continuous and amorphous phenomena whose magnitudes are dependent on their spatial locations [38]. A continuous field must be partitioned into a finite number of discrete pieces to accommodate the finite computing environment [34]. Three spatial tessellations (regular, irregular, and hybrid) are the most common means for representing field-based models. 

**Figure 1:**
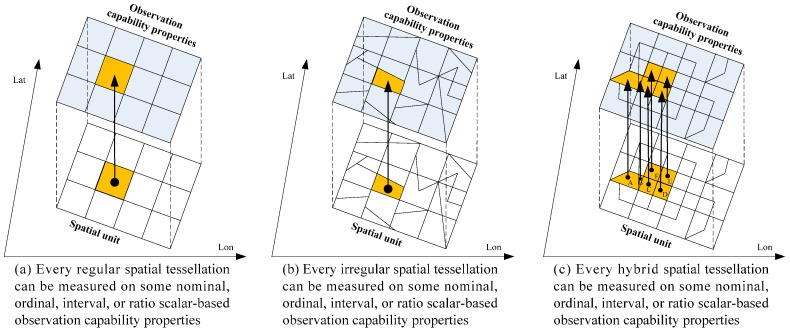
Observation capability information representation from different spatial tessellations of the field modeling perspective.

In the future, nearly every area on Earth’s surface will be observed by some physical or virtual sensors [39,40,41]. Furthermore, every section of the Earth’s surface has a potential observation request in response to a certain observation mission. Notably, the requested spatial units of an observation can be any granularity of the abovementioned three spatial tessellations and can be composed of multiple granularities of the three spatial tessellations (Figure 1). Figure 1a shows observation-requested spatial units, consisting of a set of regular spatial tessellations. Figure 1b illustrates requested spatial units, including a set of irregular spatial tessellations. The complex situation shown in Figure 1c represents spatial units composed of five hybrid spatial tessellations, where A and B are irregular tessellations and C, D, E, and F are regular tessellations. Thus, in this study, geospatial environment observation capability information varies geographically in real time, and scalar-based observation capability properties continuously surround all types of spatial units. This perspective coincides with the geospatial field modeling concept that every location in a spatial framework is associated with a set of attributes that are measured on a variety of scales.

### 2.2. Framework of GEOCF

Our living geospatial environment will be composed of ubiquitous Sensor Webs in which trillions of remote sensing and in situ sensors can be networked to create an integrated environmental monitoring system [39,40]. Specifically, in any geospatial unit, two components exist, namely, the sensors and geospatial environmental features. Based on geospatial field theory [41], the proposed GEOCF consists of a series of subfields (F_i_ (0 < i < n)) that can be expressed as Equation (1):

GEOCF = {F_1_, ..., F_m_, ..., F_j_, ..., F_n_} (1)

**Figure 2:**
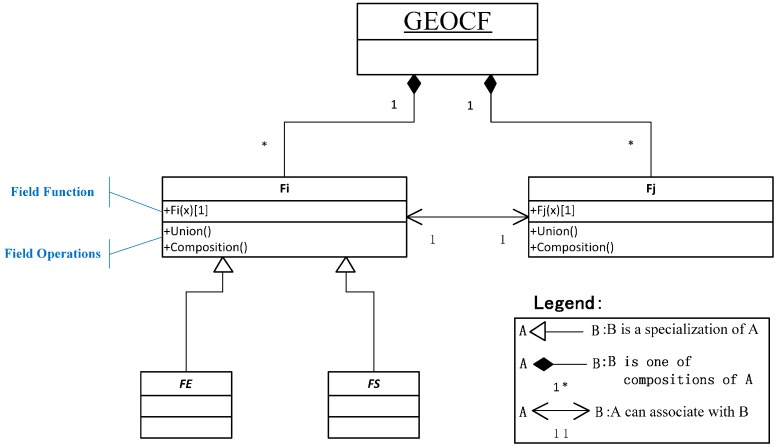
The framework of the GEOCF (Blue font represents the corresponding instructions).

Figure 2 illustrates the framework of the GEOCF model in the Unified Modeling Language (UML), which includes associations, compositions, and specializations. Each F_i_ instance encapsulates two basic elements: field function and field operations. 

As shown in Equation (2), the GEOCF can be viewed as a mapping between a geospatial framework and an observation capability domain. The formal GEOCF function can be expressed as C = f(Lon, Lat, T), where (1) Lon and Lat are every location coordinate of the continuous spatial framework; and (2) at time instant or time period T, every location in a spatial framework is associated with a set of observation capability features measured by the sets C_1_ to C_n_:

F_i_: Geospatial Framework (Lon, Lat) → Observation Capability Domain (C_1_, C_2_, …, C_n_) (2)

Each sensor establishes its sensor observation capability information field (abbreviated as FS). Due to the diversity and variety of environmental sensors [42], including numerous in situ sensors (i.e., rain gauges, water level gauges, and barometers) and the more than one thousand remote sensing sensors (i.e., Terra-MODIS, EO-1-ALI, and ENVISAT-SAR), the FS can be further represented as a set, namely {FS_1_, FS_2_, …, FS_i_, …, FS_n_}. Each FS_i_ can be viewed as the specialized subfield of F_i_. The feature components of FS_i_ can be extracted from the SensorML observation capability information representation model explained in our previous work [25], including the swath range, bandwidth ranges, and the revisit time of the sensor. The geospatial environmental features that will affect the efficiency of the sensor observation capability can form the geospatial environmental feature field (abbreviated as FE). The abstract FE is assembled from a series of subfields, such as illumination, cloudiness, and terrain fields. Namely, the FE can be represented as a set of geospatial environmental feature subfields {FE_1_, FE_2_, …, FE_i_, …, FE_n_}. 

In the proposed GEOCF, each F_i_ can be specialized into a field type (FS or FE). The field operations include union (+) and composition (○). As shown in Equation (3), if the concrete F_i_ and F_j_ are two different sensor observation capability information fields (FS_i_ and FS_j_), then the supported operation between FS_i_ and FS_j_ is a union:

FS_i_ + FS_j_: (Lon, Lat) → FS_i_ (Lon, Lat) + FS_j_ (Lon, Lat) (3)

If the concrete F_i_ belongs to FE, and the concrete F_j_ belongs to FS, the allowed operation between one instantiated field of FS (FS_i_) and one instantiated field of FE (FE_j_) is a composition (Equation (4)), which means that the practical geospatial environmental feature field FE_j_ will affect the observation capability radiated by a certain FS_i_ (herein, FE_j_ and FS_i_ have the same geographical location):

FS_i_ ○ FE_j_: (Lon, Lat) → FS_i_ (FE_j_ (Lon, Lat)) (4)

Notably, the composition operation between two geospatial environmental feature subfields (FE_i_ ○ FE_j_) has no meaning because the proposed GEOCF views FS as the original and necessary factor, whereas all components of FE simply play a constraining role in the observation efficiency of FS. 

### 2.3. Feature Components of GEOCF Information Representation Model

In actual situations, when an environmental disaster strikes in a specific geospatial unit, the availability of a sensor for the observation of this disaster is determined by: (1) time-variant, space-variant, and efficiency-variant dynamic observation capability features; and (2) sensor-inherent static observation capability features. The proposed GEOCF information representation model contains both types of observation capability features to fully meet the needs for sensor discovery and collaboration. With regard to sensor-inherent static observation capability features, our previous work [25] analyzed the observation capability metadata requirements for sharing Earth observation sensors and defined the metadata sets as a five-tuple composition: {*ObservationBreadth*, *ObservationDepth*, *ObservationFrequency*, *ObservationQuality*, *ObservationData*}. The GEOCF integrates multiple environmental sensors and considers the actual geospatial environmental features in the model. For every geospatial unit that requires observation, a real-time GEOCF is generated through a union of observation capabilities (sensor with sensor) and composition (geospatial environmental features to sensor) operations.

**Figure 3:**
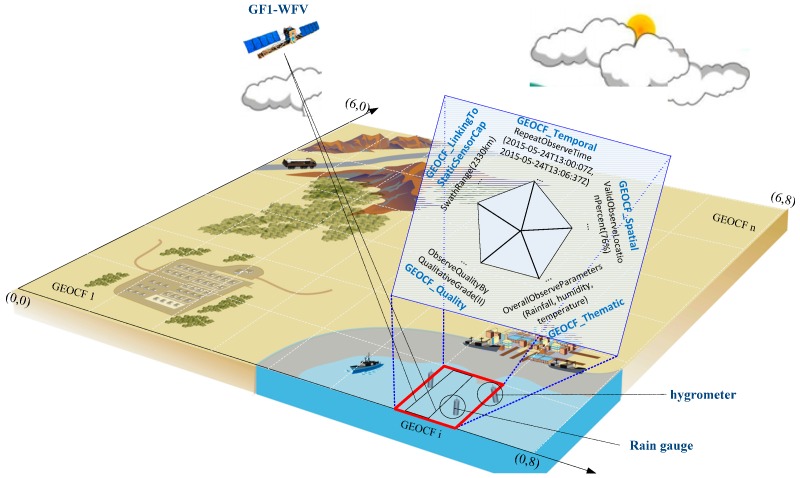
GEOCF virtual scene for environmental sensor discovery and collaboration. In the given grid cell-based geospatial observation unit (marked in red), the discovered sensors include GF1-WFV, a hygrometer, and a rain gauge; the solar elevation angle is nearly vertical, and the cloud coverage is high.

In this section, the feature components of the proposed GEOCF information representation model are identified. Figure 3 shows a virtual reality scene of the GEOCF. 

The included feature components can be interpreted as follows (Table 1).

(1) *GEOCF_Temporal*: This feature dimension identifies the period when a certain environmental disaster occurs, and sensor observation planning decisions are needed. The features regarded as *GEOCF_Temporal* features include *EachValidObserveTime*, *RepeatObserveTime*, *OverallRSObserveTime*, and *RSObserveTimePercent*.
(2) *GEOCF_Spatial*: This feature dimension refers to the spaces where an environmental disaster occurs and may include a valid, repeatedly observed, or blind observation location. Therefore, *GEOCF_Spatial* features include *EachValidObserveLocations*, *BlindObservationLocation*, *RepeatObserveLocations*, *SensorsObserveCoverageInterlinkedLocations*, and *ValidObserveLocationPercent*.
(3) *GEOCF_Thematic*: This feature dimension presents the intended applications of the available environmental sensors, including *OverallObserveParameters*, *EachSensorObserveParameters*, *ParametersInRepeatObservationLocations*, and *ParametersInInterlinkedObserveCoverageLocations*.
(4) *GEOCF_Quality*: This feature dimension is used to quantitatively and qualitatively illustrate the observation quality of the sensors, which may be affected by the geospatial environmental features in a specific geospatial unit. The features of this dimension include *ObserveQualityByQuantitativeEstimation*, *ObserveQualityByQualitativeGrade*, and *ObserveQualityBy QualitativeDescription*.
(5) *GEOCF_LinkingReference*: In addition to the dynamic observation capability features, the features of sensor-inherent static observation capabilities should be included, such as *SwathRange*, *BandsCategory*, *BandCharacteristics*, and *NadirResolution*. These features are linked from our previous representation model of Earth observation sensor static observation capability information [11].

The GEOCF is classified into three modes, namely, complementary, enhanced, and single, because different observation efficiencies will be produced when multiple sensors are combined. The efficiency of observation combination between the FS_i_ and FS_j_ can contain both complementary and enhanced modes. The complementary mode means that the observation performance of each FS_i_ in spatial and thematic applications complements one another. The enhanced mode is used to represent the scenario in which different sensors cover the same observation location and have the same observation parameter during a certain observation period. Thus, the enhanced mode refers to the FS_i_ that enhances the observation efficiency of FS_j_ in the same observation parameters in one overlapped observation area. The single GEOCF mode means that only one sensor is available for the environmental observation event in a specific geospatial location during the requested observation period. As presented in Table 1, different GEOCF modes have different feature components. The complementary GEOCF mode contains all the basic feature components. The enhanced GEOCF does not necessarily contain the exclusive features of the complementary GEOCF mode, such as *SensorObserveCoverageInterlinkedLocations* and *ParametersinInterlinkedObserveCoverageLocations*. Unlike the enhanced mode, the single GEOCF mode does not have the *RepeatObserveTime*, *LocationsWithRepeatObservation*, and *ParametersInRepeatObservation* features.

**Figure 4:**
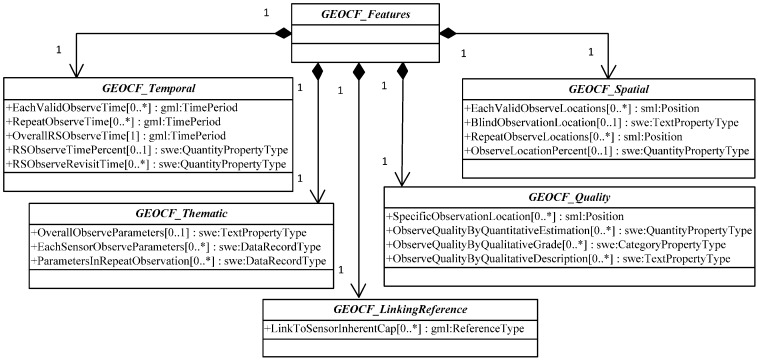
UML diagram of GEOCF feature components and their data types.

The most common geospatial field types are scalar, vector, and tensor. The proposed GEOCF is a scalar field, in which every location is assigned a scalar value from a property domain. The solution offered in the current study for the involved scalar data type is to avoid recreating the bottom data model wherever possible, but referring to the existing data models, such as Geography Markup Language 3.2, SWE Common Data Model 2.0, and SensorML 2.0. Figure 4 shows the GEOCF feature components and their data types, as well as the constraint conditions, as defined in UML. The corresponding markup language schema used to represent the GEOCF is provided at reference [43].

### 2.4. Operation Workflows of the GEOCF Information Representation Model

**Figure 5:**
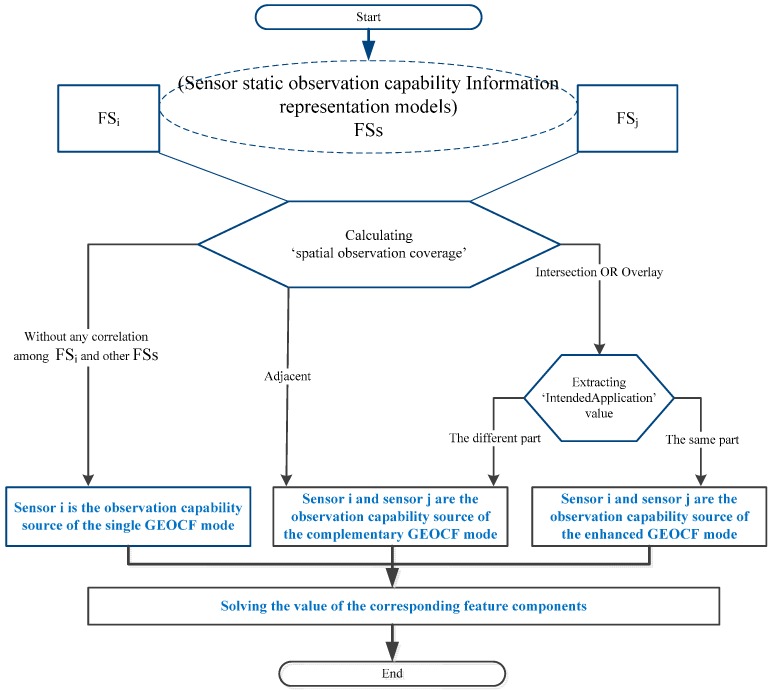
Workflow of the GEOCF union operation.

The workflow of the two operations (i.e., union and composition) is illustrated in this section. The workflow is composed of the operation input, middle processes, and output. Figure 5 shows the workflow of the union operation among different observation capability information fields (FSs) from multiple sensors. The inputs of the operation are a series of environmental sensors encapsulated in static sensor observation capability information representation model [44], to form a series of FSs. In the execution of the FS_i_ + FS_j_ operation, the spatial observation coverage of the sensors should first be determined before the cases are assessed as follows:

(1) If the spatial observation coverage of sensor i does not have any correlation with the observation coverage of the other sensors during the given requested period, then sensor i will be classified as the observation capability source in the single GEOCF mode.
(2) If the spatial observation coverage of sensor i bears a spatial relationship with the other sensor j, such as the intersection or overlay, the value for the “Sensor_designed_applications” property of sensor i and sensor j should be determined (which can be extracted from the SensorML-based static sensor observation capability information representation model). If the sensors have the same value set, *Same_ObservP* {P_k_|P_k_ ∈ FS_i_, P_k_ ∈ FS_j_}, we deem that in their intersected or overlapped observation areas, sensor i and sensor j can be combined for an enhanced GEOCF mode in the observation parameters of *Same_ObservP*.
(3) For the different value set, *Diff_ObservP* {P_k_,P_h_|P_k_ ∈ FS_i_, P_h_ ∈ FS_j_ }, sensor i and sensor j are classified as a combination of a complementary GEOCF mode in the observation themes of *Diff_ObservP*.
(4) In a special case, in which the spatial observation coverage of sensor i is spatially adjacent to that of sensor j, then sensor i and sensor j can be categorized into the complementary GEOCF mode.

In accordance with the workflows used to identify the different GEOCF modes and following the observation capability feature components of the different GEOCF modes given in Table 1, we can determine the corresponding feature component values of the entire GEOCF.

Compared with remote sensing satellite sensors that cannot be launched by an ordinary person or requester, in situ sensors can be relatively easily deployed and flexibly configured by the individual with the adjustment demand. Usually, deployers would not place in situ sensors in a geospatial environment where their observation efficiency will be obviously constrained. From the microscopic perspective, if a constraint exists, the deployer could configure additional in situ sensors on demand to as much as possible compensate for the observation efficiency of a single in situ sensor constrained by the local geospatial environment. Unlike in situ sensors, satellite sensors are used to observe an object and obtain information from a great distance by detecting the radiant energy reflected or emitted by the object. For passive satellite sensors, stereoscopic space, which consists of topography, cloudiness, and the sun, affects the radiation transmitted from the ground to the sensor when observing the Earth. Topography is the main factor that affects radiation for active satellite sensors. With the above analysis, our current study focuses on the observation effect of remote sensing satellite sensors by the geospatial environmental conditions. The concrete instantiations of FE are illumination, cloudiness, and terrain fields. The FS sensor types are passive optical and active synthetic aperture radar (SAR) satellite sensors. 

**Figure 6:**
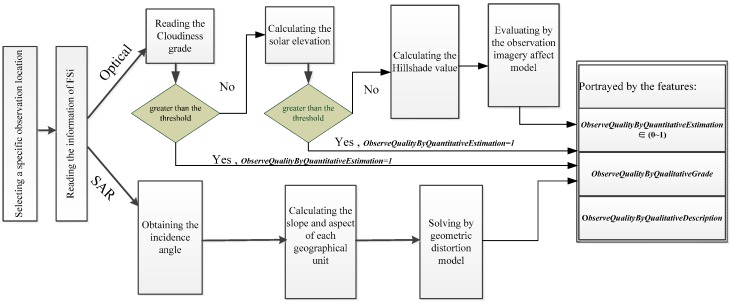
Workflow of the GEOCF composition operation.

Figure 6 demonstrates the workflow of the FS_i_ ○ FE_j_ operation. A specific location is first selected, and the observation quality herein must be measured for the applicability estimation of the discovered sensor. Then, according to the FS sensory types, the observation efficiencies of the optical satellite sensors are affected by the geospatial environmental features (e.g., cloudiness grade, which can be read from the cloud field; solar elevation from the illumination field; and hill shade from the terrain field); and the observation efficiencies of the SAR sensors are affected by the slope and aspect, which can be read from the terrain field. The final observation quality is represented by three features: *ObserveQualityByQuantitativeEstimation*, *ObserveQualityByQualitativeGrade*, and *ObserveQualityByQualitativeDescription*. Equation (5) referenced from the existing observation imagery affect model [45] exemplifies the calculation function of the *ObserveQualityByQuantitativeEstimation* value of the passive optical satellite sensors:

*I_r_* = (*I_d_* × *dx* + *I_s_*)(1 − *CC*/10)^2^ (5)

where *I_r_* is the *ObserveQualityByQuantitativeEstimation* value; *I_d_* represents the theoretical energy estimation that the ground has received from the direct solar radiation; *I_s_* represents the real energy estimation that the ground has received from the solar diffuse radiation, which is affected by the solar zenith angle and atmospheric transparency; *dx* is the terrain factor; and *CC* is a cloud-level value ranging from 0 to 10. The *ObserveQualityByQualitativeGrade* is a qualitative indicator used to describe the grade of observation quality affected by the geospatial environmental factors, which will be divided according to the *ObserveQualityByQuantitativeEstimation* values. The *ObserveQualityByQualitativeDescription* feature is used to illustrate the corresponding relationship between the quantitative estimation value and the qualitative grade by a text description. 

## 3. Sensor Discovery and Planning Experiment of Flood Observation in the Jinsha River Basin

### 3.1. Flood Observation of the Jinsha River Basin

#### 3.1.1. Flood Observation Requirement

**Figure 7:**
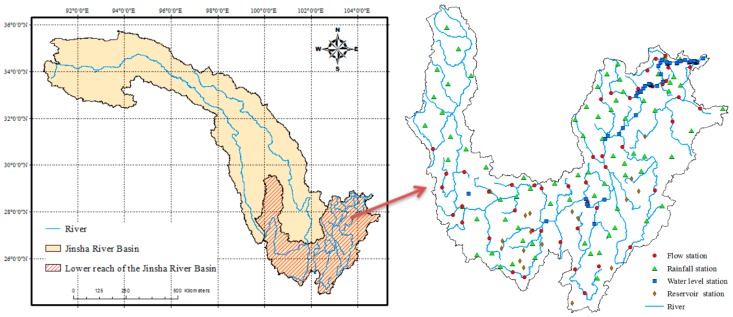
Location of the study area.

The Jinsha River Basin is located in the upper reaches of the Yangtze River and has a total length of 3464 km [46]. The annual precipitation in the basin exceeds 1600–2000 mm. The Jinsha River Basin can be divided into upper, middle, and lower sections. As shown in Figure 7, the lower section (latitude: 24°59′26″ N–29°37′16″ N, longitude: 99°47′48″ E–104°33′42″ E) begins in Panzhihua City and ends in Yibin City. The basin has a length of 733.4 km and a watershed area of 135,473 km^2^. The terrain is highly elevated in the west and low in the east, creating a ladder-level distribution. The runoff from the Jinsha River Basin is flushed from north to south, and the lower section is the most vulnerable to flooding because of heavy rainfall in the upper section. The flow of the basin is variable and exhibits a seasonal behavior. The flow is low during winter months, and the peak flow occurs in May and October. 

#### 3.1.2. Existing Sensor Resources

As drawn from the Committee on Earth Observation Satellites (CEOS) system database [47], 137 satellite sensors are currently supporting flood observation. In the lower reaches of the Jinsha River Basin, more than 100 in situ hydrology, meteorology, and soil monitoring sensors have been deployed (Table 2). The current study has constructed the SensorML-based static observation capability information representation models of more than 200 sensors based on our previous SensorML modeling method [48]. Through the CSW interface, these sensors have been registered and published in the flood sensor clearinghouse of the Jinsha River Basin to be the sensor library used in this case.

### 3.2. Realistic Problem before Using the GEOCF as the Information Foundation

The targeted monitoring space (Jinsha River Basin) is a wide area, and flood forecasting models and observation themes often involve more than one observation parameter. In addition, the candidate sensors in the area have been deployed and managed by different administrative departments. Resultantly, one person cannot determine which sensors can collaboratively meet the observation request. In the event of future flood observations, questions such as “how can we comprehensively plan suitable sensors for this task?” and “can sensor A and sensor B enable an enhanced effect in the observation of theme I?” must be answered. Because of the mutually independent SensorML-described observation capability information, the sensor discovery and planning decision-makers face a tedious and time-consuming process of exploring the associated observation capability properties from a massive amount of SensorML-based observation information models to determine which sensor combination is the most rational solution. That is, by using the previous SensorML observation information model and OGC SWE-based sensor query systems, decision-makers will obtain a list of matched sensors in the specified observation mission. However, decision-makers cannot grasp the correlated observation capability information. Thus, decision-makers will not know how to use those sensors for collaboration effectively.

To select the sensors or combination of sensors that can most effectively meet the complex flood observation request, decision-makers must acquire reliable and comprehensive observation capability information. Therefore, the realistic significance of this work is that the proposed GEOCF can help the sensor inquirer answer questions such as “how can we combine these sensors in their suitable observation time and space to maximally meet the given observation mission?” “what is the union observation efficiency between sensor A and sensor B?”, and “how much of the observation influence of the MODIS sensor equipped on the AURA satellite is affected by the local geospatial environment features?” 

### 3.3. Flood Sensor Discovery and Planning in GEOCapabilityManager 

From a software developing perspective, performance engineering [49] is a discipline that attempts to integrate concerns about the feasibility, availability, and security of software applications and their satisfaction degree into user requirements of the real application. A prototype system, GEOCapabilityManager, is designed to implement the related observation experiment. The performance metrics used to measure the efficiency of the proposed GEOCapabilityManager are important. The GEOCapabilityManager of the current stage supports the modeling of the integrated and dynamic observation capability information and planning the matched multiple sensors for monitoring because our study focuses on representing the GEOCF features. Some important quantitative performance metrics, such as the responsiveness of the GEOCapabilityManager, the accuracy of the GEOCF application and the satisfaction degree of the planning decision, will be considered in the later stage of the GEOCapabilityManager development. 

**Figure 8:**
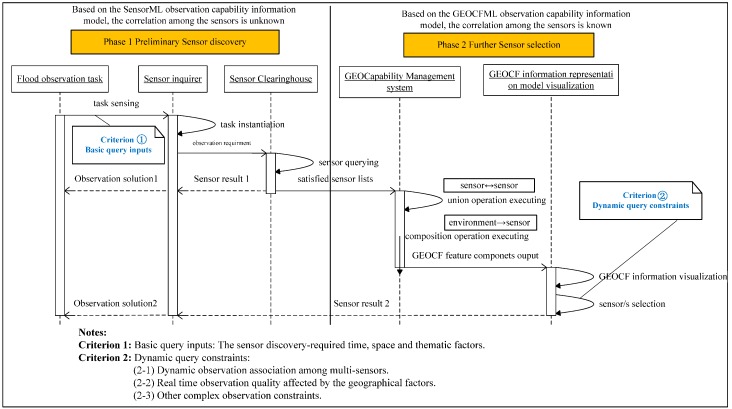
UML sequence diagram showing sensor discovery with the GEOCF information representation model.

The procedure for flood sensor discovery and planning in the GEOCapabilityManager system is shown in Figure 8, which includes two phases: preliminary sensor discovery and further sensor selection. 

In Phase 1, the sensor inquirer should first identify the basic observation query criteria, including the sensor discovery-required time, space, and thematic factors for the actual flood observation task. Then, the targeted sensors will be preliminarily matched from the flood sensor clearinghouse according to the given observation query requirements. The sensor list from this phase is composed of multiple sensors that are not correlated to one another. Phase 2 involves executing the core functions of our developed GEOCapabilityManager system, including (1) the union operation among the matched sensors; and (2) the composition operation between the geospatial environmental feature and the sensor. The composition operation will be triggered if the sensor inquirer determines whether or to what extent the sensor observation quality is affected by the local geospatial environmental features. After these two operations, the value of the GEOCF feature components can be identified. In the last visualization stage, the GEOCF information, including the correlated observation capability information of these matched sensors and the observation quality information affected by the geospatial environmental features, will be explicitly represented in GEOCFML description language. Sensor inquirers can map the real dynamic query constraints criteria to the GEOCFML-described information model, and generate the sensor observation planning program marked as “Observation solution 2” in which we can obtain the associated sensor relationship and sensor observation quality information, more so than the “Observation solution 1” (marked in Figure 8), which only returns a list of available sensors.

#### 3.3.1. Basic Flood Observation Query

**Figure 9:**
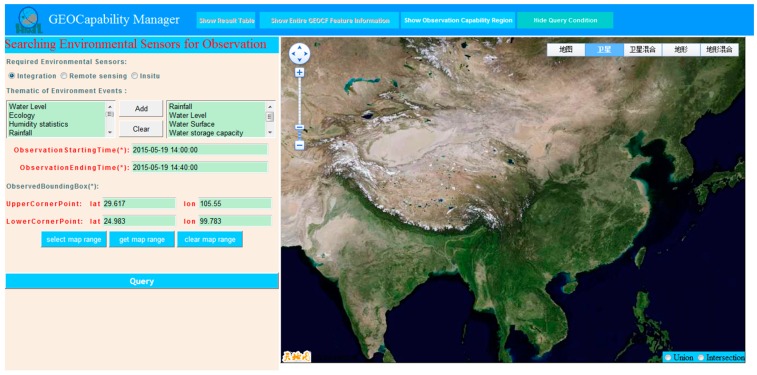
The observation query mission input into the GEOCapabilityManager.

Flood monitoring is a complex process comprising several stages [46]. Every stage has a set of diverse and complex flood forecasting models. Regardless of how complex the observation scenario is, it can be decomposed into sub-observation segments; regardless of how integrated the flood forecasting model is, it can be assigned to some specific observation parameters. The observation query entrance of our developed GEOCapabilityManager system applies the Pareto principle (also known as the 80:20 rule) and aims to describe most observation requirements with the least complexity. Intentionally, it does not support every detail of an observation requirement but concentrates on the common features shared by a wide range of sensor inquirers. That is, although different decision-makers have different observation query requirements, we only use the basic input of the observation task as the observation query requirements, including the sensor discovery-required time, space, and thematic factors. Therefore, in this actual flood observation application, we set the query mission where the observation time, space, and thematic requirements are as follows (Figure 9): start time is 2015-05-19T14:00:00; end time is 2015-05-19T14:40:00; spatial unit (the lower reaches of the Jinsha River Basin) is between 24.938° N and 29.617° N and between 99.783° E and 105.55° E; and required observation parameters are water level, rainfall, silt, water surface, water storage capacity, and multipurpose imagery (land).

**Figure 10:**
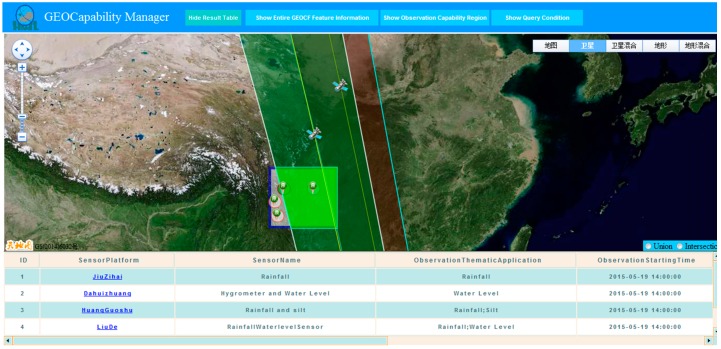
Discovered sensors for the given flood observation task.

Figure 10 shows the result of the search request. From the current sensor library, six qualified environmental sensors, namely, MODIS-AURA, AEISS-KOMPSAT 3A, Rainfall-JiuZihai, WaterLev-DaHuiZhuang, Rainfall-LiuDe, and Rainfall & Silt-HuangGeShu, are targeted. These sensors are tagged with serial numbers.

#### 3.3.2. Considering Multi-Sensors’ Union

**Figure 11:**
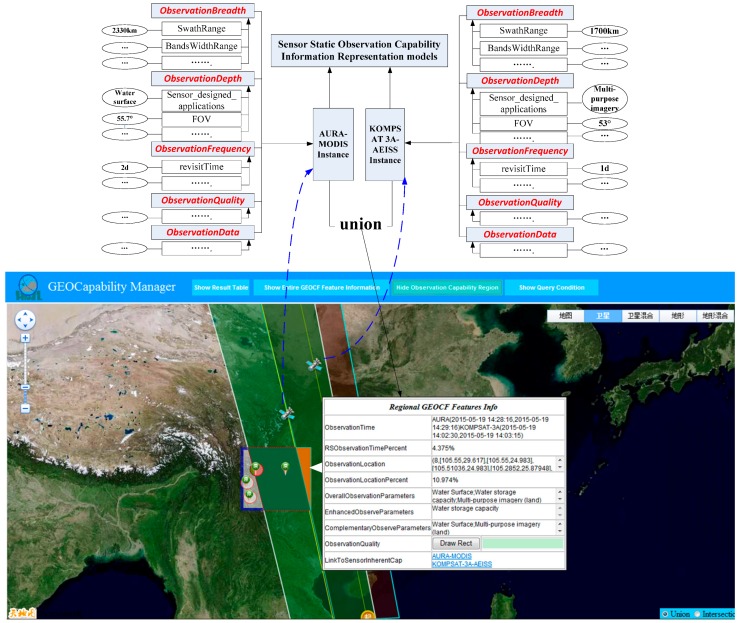
A sample of the sensor–sensor union operation in regional GEOCF modeling. The displayed regional GEOCF feature information refers to the integrated observation capability of the overlapping observation coverage (filled in red) between MODIS-AURA and AIESS-KOMPSAT 3A.

The “sensor ↔ sensor union” and “geospatial environmental features → sensor composition” operations should be executed to construct the proposed GEOCF. Following the workflow of the union operation described in Section 2.4, two satellite sensors, namely, MODIS-AURA and AEISS-KOMPSAT 3A, are selected to verify the GEOCF modeling in the temporal, spatial, and thematic dimensions. In this union experiment (Figure 11), the existing SensorML-based sensor static observation capability information representation models serve as bases to formulate a series of FSs from where the feature components (e.g., inherent satellite sensor ID, sensor associated platform, sensor measure type, and field of view) can be extracted to calculate the dynamic satellite orbit and observation coverage of the satellite sensors. Then, the intersection of the sensors’ observation coverages can be determined. Whether two sensors have the same observation parameters in the intersection observation location should be determined by further extracting the value of “Sensor_designed_applications” from the FSs. As shown in Figure 11, the regional GEOCF results from the union operation between MODIS-AURA and AIESS-KOMPSAT 3A exhibit some dynamic observation capability information, such as: the overlapping observation coverage (filled in red) between MODIS-AURA and AIESS-KOMPSAT 3A is 10.974% of the observation-requested spatial extent, and three observation parameters can be completed in the overlapping observation coverage. The two sensors, AURA_MODIS in time period (2015-05-19T14:28:16Z, 2015-05-19T14:29:16Z) and KOMPSAT-3A_AEISS in time period (15-05-19T14:02:30Z, 2015-05-19T14:03:15Z), have the same observation parameter in “Water storage capacity” in the overlapping observation coverage which is surrounded by eight observation points (105.55° E, 29.617° N), (105.55° E, 24.983° N), (105.35857° E, 24.983° N), (105.23668° E, 25.47171° N), (104.99878° E, 26.41133° N), (104.75719° E, 27.35069° N), (104.51174° E, 28.28979° N) and (104.15718° E, 29.617° N). The two sensors can formulate an enhanced observation efficiency of “Water storage capacity” and a complementary efficiency in “Water Surface” and “Multi-purpose imagery (land)”, which clearly shows the associated observation efficiency of two independent sensors. The inherent observation capability information of the two sensors can also be viewed via the hyperlinks in the SensorML-based observation capability information representation model.

#### 3.3.3. Considering the Effect of Geospatial Environmental Feature Factors

**Figure 12:**
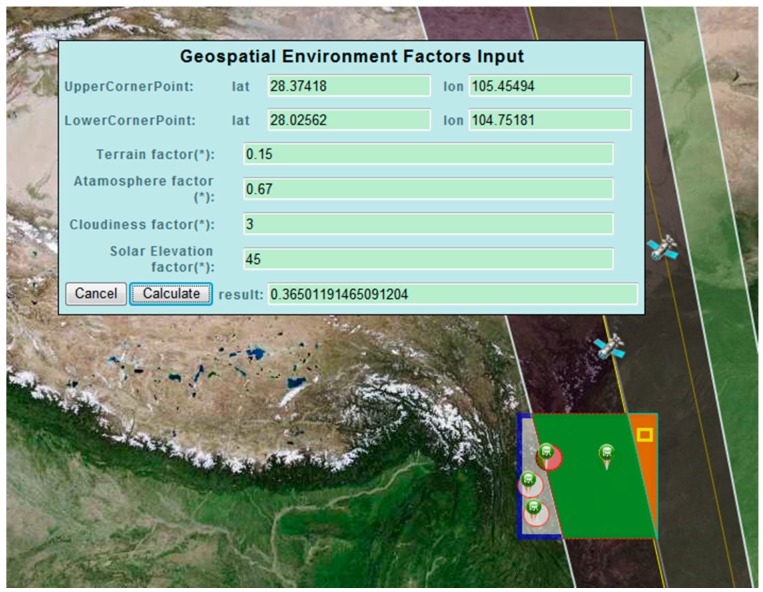
Geospatial environmental features inputted for the observation quality calculation. Notably, the smaller the selected geospatial unit is, the closer the calculated result is to the actual environmental feature value, thus facilitating a more accurate evaluation of the observation quality.

In the composition operation experiment, an observation area should be first selected where the sensor observation quality will be evaluated. As shown in Figure 12, a specified observation area (marked in yellow) is selected from the overlapping observation coverage of two satellite sensors. Then, the environmental feature factors are manually input. The first environmental feature is the terrain factor, which can be solved using the hill shade analysis function of the ArcGIS tool. The second feature is cloud coverage grade, which can be read from the Open Weather Map website [50]. The third feature is atmospheric transparency, which is obtained using an on-site atmospheric transmission instrument. The fourth feature is the solar elevation angle, which is the angle between a line from the sun to the imaging point of the Earth’s surface and the local horizontal plane. The corresponding values of the four environmental features are presented in Figure 12. The two discovered satellite sensors are optical sensors; thus, we comply with the workflow to solve the effect of geospatial environmental features on the observation efficiency of the two optical satellite sensors. The quantitative observation quality estimation result is shown in Figure 12. 

#### 3.3.4. Entire GEOCF Represented in a Uniform Information Model

**Figure 13:**
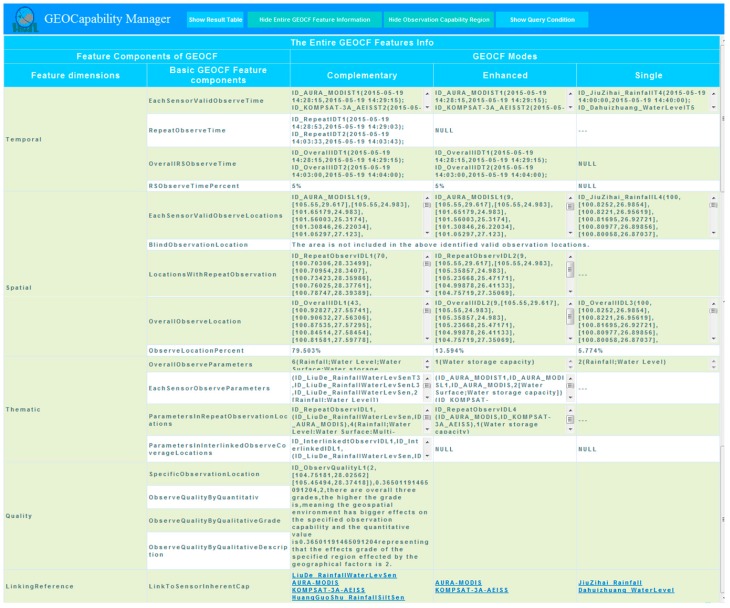
A sample table representing all GEOCF feature components. This is an instance of Table 1.

The visualization module of the GEOCapabilityManager can facilitate (1) the browsing of the regional GEOCF feature components generated after the union of certain sensors (Figure 11) and the composition effect of the geospatial environmental features on the sensors (Figure 12); and (2) the viewing of the entire GEOCF feature components (Figure 13), which comprehensively consider all the retrieved environmental sensors and the environment feature factors. For example, Figure 13 shows the relationships of observation capabilities of multiple sensors with the use of a table, from which the following can be identified: (1) In the observation area (*ID_RepeatObservIDL1*), the union of sensor i (*ID_LiuDe_RainfallWaterLevSen*) and sensor j (*ID_AURA_MODIS*) allows for a complementary observation of parameters such as “Rainfall”, “Water Level”, “Water Surface”, “Multi-purpose Imagery (land)”, and “Water storage capacity”; (2) In the overlapping observation spatial area (*ID_RepeatObservIDL2*), the union of sensor i (*ID_AURA_MODIS*) and sensor j (*ID_KOMPSAT-3A_AEISS*) allows for an enhanced observation of the “water storage capacity” parameter; (3) In the selected observation location (*ID_ObservQualityL1*), the composition of the two satellite sensors and the geospatial environmental features demonstrate that the geospatial environmental features influence the observation efficiency of the satellite sensors at a quantitative observation quality estimation value of 0.3650119465091204. This value indicates that the effect grade of the specified region affected by geographical factors is II. (There are three grades overall; the higher the grade is, the greater the effect of the geospatial environment is on the specified sensor observation capability).

**Figure 14:**
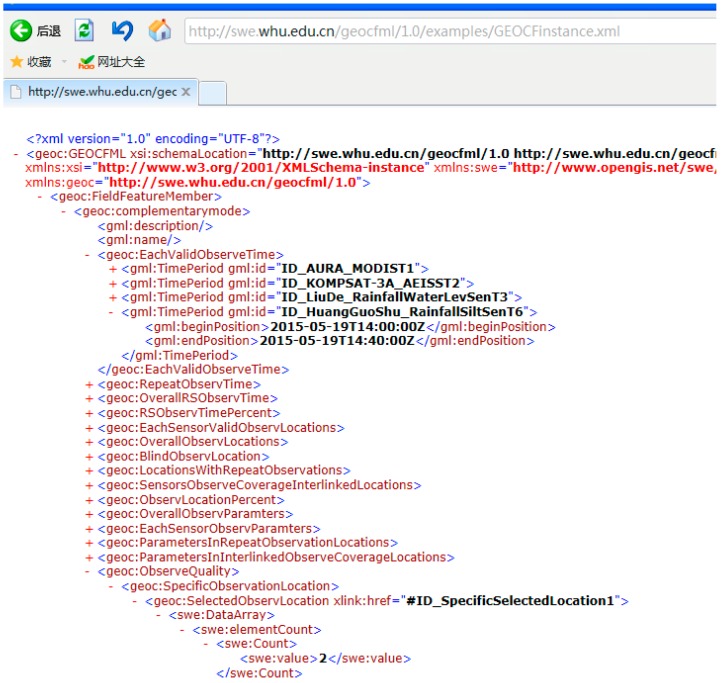
A sample of the GEOCF information representation model.

We use the GEOCF markup language to formalize the GEOCF. Figure 14 shows a segment of the solved GEOCF information representation model instance. The complete record of the instance can be viewed at reference [51]. In conclusion, the integrated observation capability information of all the sensors in a specific observation-needed geospatial environment can be obtained from the constructed GEOCF information representation model.

## 4. Discussion

### 4.1. Comparison to the SWE Sensor Observation Capability Information Model

Past SWE SensorML-based observation capability information representation starts from the perspective of a sensor or sensor system object. The observation capability properties of each object are formalized into an information representation model. Specifically, 100 sensor systems have 100 sensor observation capability information representation models. No description of the association of observation capability among those sensors is available. The proposed GEOCF is regarded as a mapping between geospatial locations and observation capability features. It links the sensors located in a specific observation space and considers the influence of real-time geospatial environmental features on sensor observation capability. Our GEOCF can be formalized by the GEOCF markup language (GEOCFML); the GEOCFML-based observation capability information can be easily read and archived by an inquirer on demand. Unlike the previous SensorML-based information representation model, which only records single-sensor static observation capability information and disregards the constraints from geospatial environmental features, the GEOCFML-based information representation model records the associated observation capabilities of multiple sensors and the dynamic observation quality impacted by the local geospatial environmental conditions.

Using six qualified sensors displayed in Figure 10 as examples, before executing the proposed field operations (union and composition) illustrated in Section 3.3.2 and Section 3.3.3, those sensors are only discrete objects, and the correlation among them is unknown. The planning process of environmental sensor collaboration requires integrated and dynamic observation capability information in different dimensions including temporal, spatial, thematic, and observation quality, all of which have been covered in our proposed GEOCF. As seen from the given instance [51], we can obtain the observation association from different sensors and the quantitative observation quality estimation of AURA_MODIS affected by the geospatial environmental conditions. 

Every section of the Earth’s surface is a potential geospatial unit that requires observation, which can be any granularity of the spatial tessellations described in Figure 1. From the observation capability cognitive perspective, the GEOCF provides an important index for comprehensively understanding the objective, dynamic, and real-time associated observation capability information of any space requiring observation in the geospatial environment. By contrast, SensorML-based sensor observation capability information provides an understanding of the inherent and static observational nature of one sensor.

In conclusion, the GEOCF information representation model is an extension of the SWE SensorML-based sensor observation capability information model. This model is a logical and integrated observation capability information model that can record the associated observation capability information among multiple sensors and estimate sensor observation quality affected by geospatial environmental factors. It can play a major role in observation capability sharing and assist in the integrated management of the available flood observation sensors.

### 4.2. Comparison with Existing Sensor Discovery and Planning Systems

Although various systems or tools can be used for sensor discovery or planning, they are characterized by low reliability and fails to support environmental sensor collaboration. Table 3 shows the characteristics of our proposed GEOCapabilityManager in comparison with eight sensor management tools or systems from different aspects. The NASA Global Change Master Directory retrieval portal [52] and other public search engine sites (such as Google and Yahoo) are used to search for sensors. The ambiguous mode of entering “free text” and “filter list” as query criteria is adopted. The Remote Sensing Planning Tool [53] provides a simulation of the dynamic trajectories of satellite sensors but can only be used for the planning of satellite sensors. The CEOS database [54] and the World Meteorological Organization observing systems capability analysis and review tool [55] provide statistics on in-orbit satellite sensors and demonstrate their static capability to assist sensor inquirers. Geosensor [56] is a Web-based sensor system that relies on the 52° North open source package [57] to facilitate environmental observation planning and access. The Sensor Instance Registry [58] is a Web service interface for managing the metadata and status information of sensors, allowing for a sensor inquirer to search for sensor instances based on static observation capability metadata. These systems establish the general information or static observation capability information representation model from an object of a single sensor. Different systems have different representation models. Furthermore, sensor schedule reliability cannot be guaranteed because the existing sensor information representation models do not consider geospatial environmental features as influencing factors of sensor observation capability. However, our GEOCapabilityManager can support the evaluation of observation quality influenced by geospatial environment features (Figure 12).

### 4.3. Extension to Other Environmental Observation Applications

All environmental disaster events require integrated and dynamic observations to monitor the different environmental phenomena in the developmental stages of a disaster. For example, in landslide monitoring, variables such as rainfall, soil moisture, surface deformation, slope, aspect, and land cover type must be measured. To observe these variables, some decision-makers may require complementary observations in the same period over a continuous space, whereas others may require enhanced observation in the same location at specific time intervals. In the initial stages of landslide incubation, rainfall, soil moisture, and land cover of the potential landslide surface should be extensively monitored. These variables can be complementarily observed with a rain gauge, a hygrometer, and satellite sensors. However, during the imminent triggering of a landslide, an enhanced observation of the soil moisture of a specific location at one time (e.g., time instant 1 by LANDSAT-MSS and time instant 2 by SPOT5-CCD) is needed. Thus, a landslide forecast can be considered an observation scenario that requires dynamic observation capabilities described in an integrated model framework, rather than static observation capabilities described by a single sensor. Currently, owing to the underutilization of environmental sensors, decision tools for discovering credible environmental sensors and planning the collaboration of these sensors in an actual observation application are not yet available. The proposed GEOCF is capable of: (1) dynamically organizing the observation capabilities of two or more environmental sensors previously deemed independent of one another; (2) accurately evaluating the credibility of available sensors; and (3) rationally scheduling the various environmental sensors to satisfy observation requirements. Thereby, it can assist in answering the question “Which sensor combination can be selected to measure the required variables in the given observation time and space?” and be extended to other environmental observation applications.

## 5. Conclusions and Outlook

This study introduces a Field-based Geospatial Environmental Observation Capability (GEOCF) information representation model that comprises field function, field feature components, and field operations. The effectiveness of the proposed GEOCF information representation model is verified by applying it to sensor discovery and collaboration for flood observation of the lower reaches of the Jinsha River Basin. Results confirm that our proposed model can perform as: (1) an integrated descriptor for comprehensively understanding geospatial environmental observation capability information; (2) an information foundation that promotes the reliable utilization of environmental sensors and scheduling of environmental sensor collaboration for flood observation; and (3) a feasible source to be extended to other environmental observation applications. 

Our proposed GEOCF mainly considers the geospatial environmental factors that will affect the sensor observation quality. Therefore, other environmental dynamics, such as the dynamic monitoring object and the surface feature distribution, should be considered in our next study to construct a more accurate GEOCF information representation model. In addition, the functions of autonomous accessing the extracting the geospatial environmental factors will be integrated into our prototype to form an intelligent system. The theory of performance engineering should be reflected in the follow-up design and implementation of GEOCF information representation model.

## Figures and Tables

**Table 1:** Feature components of the GEOCF in different field modes.

The Entire GEOCF Features Info				
Feature Components of GEOCF Information Representation Model		GEOCF Modes		
Feature Dimensions	Basic GEOCF Feature Components	Complementary	Enhanced	Single
Temporal	*EachSensorValidObserveTime*	√	√	√
	*RepeatObserveTime*	√	√	×
	*OverallRSObserveTime*	√	√	√
	*RSObserveTimePercent*	√	√	√
Spatial	*EachSensorValidObserveLocations*	√	√	√
	*BlindObservationLocation*	√	√	√
	*LocationsWithRepeatObservation*	√	√	×
	*SensorsObserveCoverageInterlinkedLocations*	√	×	×
	*OverallObserveLocations*	√	√	√
	*ObserveLocationPercent*	√	√	√
Thematic	*OverallObserveParameters*	√	√	√
	*EachSensorObserveParameters*	√	√	√
	*ParametersInRepeatObservationLocations*	√	√	×
	*ParametersInInterlinkedObserveCoverageLocations*	√	×	×
Quality	*SpecificObservationLocation*	√	√	√
	*ObserveQualityByQuantitativeEstimation*			
	*ObserveQualityByQualitativeGrade*			
	*ObserveQualityByQualitativeDescription*			
LinkingReference	*LinkToSensorInherentCap*	√	√	√

**Table 2:** List of the hydrometeorological stations in the lower reaches of the Jinsha River Basin used in this study.

Station ID	Station Name	Longitude (°E)	Latitude (°N)	Observe Parameters	Administrative Department
60405250	DeZe	103.598889	25.993333	Water Quality	Yunnan Provincial Hydrology Bureau
60407110	HengJiangQiao	104.411585	28.613476	Evaporation	South Central Survey and Design Institute
60426800	QingNian	103.015833	25.203889	Reservoir silt	Yunnan Provincial Hydrology Bureau
60102525	WuDongDe	102.622822	26.299007	Flow, flow rate	Changjiang Water Resources Commission
60224950	LiuDe	101.0063889	26.484444	Rainfall	Yunnan Provincial Hydrology Bureau
……					

**Table 3:** Comparison between existing sensor management systems and GEOCapabilityManager.

Aspects	Tools or Systems for Sensor Planning and Discovering Management						
	GCMD	Google/Yahoo	RESPT	WMO/CEOS	Geosensor	SIR	GEOCapability Manager
Sensor object	Remote sensing & in-situ sensors	All types of sensors	Remote sensing satellite sensors	Remote sensing sensors	Remote sensing & in-situ sensors	In-situ sensors	Remote sensing & in-situ sensors
Main usage	Sensor searching	Sensor researching	Sensor planning	Sensor capability review	Sensor observation discovery & service	Sensor discovery	Sensor discovery & planning
Sensor Modeling mode	Single & Static sensor	Single & Static sensor	Single & dynamic sensor	Single & Static sensor	Single & Static sensor	Single & Static sensor	Multiple & Dynamic sensors
Representing format	Html text	N/A	N/A	text	SensorML	SensorML	GEOCFML
Supporting Multi-sensors collaboration	NO	NO	NO	NO	NO	NO	YES
Considering the geospatial environment features	NO	NO	NO	NO	NO	NO	YES
